# The *UDP-glucose ceramide glycosyltransferase* (UGCG) and the link to *multidrug resistance protein 1* (*MDR1*)

**DOI:** 10.1186/s12885-018-4084-4

**Published:** 2018-02-06

**Authors:** Marthe-Susanna Wegner, Lisa Gruber, Peter Mattjus, Gerd Geisslinger, Sabine Grösch

**Affiliations:** 1 pharmazentrum frankfurt/ ZAFES, Institute of Clinical Pharmacology, Johann Wolfgang Goethe-University, House 74, Theodor Stern-Kai 7, 60590 Frankfurt am Main, Germany; 2 Biochemistry, Faculty of Science and Engineering, Åbo Akademi University, Artillerigatan 6A, III, BioCity, FI-20520 Turku, Finland

**Keywords:** UDP-glucose ceramide glycosyltransferase, UGCG, Glycosphingolipids, MDR1, P-gp, Multidrug resistance, Cancer

## Abstract

The UDP-glucose ceramide glycosyltransferase (UGCG) is a key enzyme in the sphingolipid metabolism by generating glucosylceramide (GlcCer), the precursor for all glycosphingolipids (GSL), which are essential for proper cell function. Interestingly, the UGCG is also overexpressed in several cancer types and correlates with *multidrug resistance protein 1* (*MDR1*) gene expression. This membrane protein is responsible for efflux of toxic substances and protects cancer cells from cell damage through chemotherapeutic agents. Studies showed a connection between UGCG and *MDR1* overexpression and multidrug resistance development, but the precise underlying mechanisms are unknown. Here, we give an overview about the UGCG and its connection to *MDR1* in multidrug resistant cells. Furthermore, we focus on UGCG transcriptional regulation, the impact of UGCG on cellular signaling pathways and the effect of UGCG and *MDR1* on the lipid composition of membranes and how this could influence multidrug resistance development. To our knowledge, this is the first review presenting an overview about UGCG with focus on the relationship to *MDR1* in the process of multidrug resistance development.

## Background

Over the last two decades, researchers were able to link the sphingolipid metabolism and the development of multidrug resistance in several cancer types, but the precise mechanisms are unknown. There is evidence that the *UDP-glucose ceramide glycosyltransferase* (UGCG), which is located in the Golgi apparatus (Fig. 4) is involved in the process of multidrug resistance development. The UGCG was first cloned by *Ichikawa* et al. in the year 1996 [1] and is currently the only enzyme responsible for the de novo production of glucosylceramide (GlcCer). GlcCer is the precursor for all glycosphingolipids (GSLs) such as globo-series and ganglio-series GSLs. Since more than 400 species of GSLs are identified, the GSL metabolic pathway is here described only briefly (reviewed in [2]). Ceramides, generated in the endoplasmic reticulum by ceramide synthases (CerS), are transported to the Golgi apparatus, where the UGCG transfers UDP-glucose to ceramide resulting in GlcCer. Subsequently, the produced GlcCer is flipped from the cytosol to the Golgi apparatus lumen for lactosylceramide (LacCer) production by galactosylation of GlcCer. LacCer serves as a substrate for a variety of GSLs producing enzymes (reviewed in [2]). From the Golgi apparatus GSLs are transported to the plasma membrane, where they are integrated (Fig. 4). GSLs depletion in the plasma membrane is executed by endocytotic membrane flow of GSLs into the lysosomes, where glycohydrolase activity results in GSLs degradation and subsequently ceramide production [3]. GSL are essential for embryonic development and differentiation of several tissue types [4] and are involved in lipid storage disorders called sphingolipidoses, which are caused by deficiency of lysosomal enzymes. One of the main sphingolipidoses is the Gaucher disease, which is characterized by deficiency of glucocerebrosidase leading to GlcCer accumulation in cells and organs. Due to different phenotypes the clinical presentation of Gaucher disease patients ranges from cytopenia to neurological impairment (reviewed in [5]). In patients with Fabry disease an GSL accumulation (in general glycolipids) occurs also, but in this case due to mutations in the *GLA* gene encoding α-galactosidase A (reviewed in [6]). The Sandhoff and Tay Sachs disease are characterized by accumulation of the GSL GM2 leading to neurodegeneration and early death (reviewed in [7]). Although, the phenotype of the Niemann-Pick disease is based on deficient sphingomyelinase activity, GlcCer concentration is found to be the second highest lipid concentration [8]. The different types of sphingolipidoses show how important it is to understand the sphingolipid metabolism and how complex it is. To date, only few reviews dealing with the UGCG are available [9–11], therefore we will discuss the impact of the UGCG and its knockout on cell proliferation and cell metabolism shortly.

**Fig. 1:**
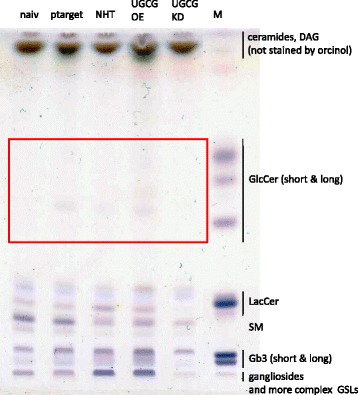
High-performance thin-layer chromatography (HPTLC) analysis of diverse sphingolipid species in MCF-7 cells. Representative HPTLC plate showing the separation of ceramides, DAG and non-complex GSLs like GlcCer, LacCer and complex GLSs like Gb3 and gangliosides. MCF-7/naiv: untransfected, MCF-7/pTarget: empty vector-transfected, MCF-7/NHT: CRISPR-Cas non-human target control, MCF-7/UGCG OE: UGCG expression plasmid-transfected, MCF-7/KD: CRISPR-Cas UGCG Knockdown. M = Marker

A knockout of the UGCG in mice leads to embryonic lethality, which occurs at the phase of gastrulation. However, *Yamashita* et al. showed a not discriminable proliferation rate between UGCG deleted embryonic stem cells and wildtype cells in vitro, although GSL synthesis was absent in the UGCG disrupted embryonic stem cells [4]. Instead, nude mice studies showed that silencing the UGCG gene in adriamycin-resistant MCF-7 cells, which overexpress the UGCG, inhibits tumor xenograft growth in vivo [12]. This result is in line with the studies of the Sabatini Lab (Whitehead Institute, Cambridge, Massachusetts) showing that the UGCG is an essential protein for optimal proliferation of several cancer cell lines [13]. The working group developed a *Clustered Regularly Interspaced Short Palindromic Repeats* (CRISPR) score (CS), which mirrors the loss of fitness evoked by knockout of the respective gene. The CS is represented „as the average log2 fold-change in the abundance of all sgRNAs targeting a given gene” and indicates whether the gene knockout leads to an increased or decreased cancer cell number. For the UGCG the following CS were determined: KBM7 (− 0,243), K562 (0,163), Jiyoye (− 0,08) and Raji (0,364) cells [13]. These values show that the UGCG is essential for cell proliferation, but how severe the impact on the cell metabolism is seems to be cell-type specific. In summary, contradictory results were obtained regarding the essentiality of the UGCG gene for cell proliferation. Another explanation for the contradictory results could be the existence of a second form of this protein, which balances the loss of the other protein. But our high-performance thin-layer chromatography (HPTLC) studies show that there is no compensation of GlcCer loss in UGCG deficient MCF-7 cells (lane 5) (Fig. 1). Therefore, UGCG may be a unique protein in cells, solely responsible for the first glycosylation step of ceramides leading to complex GSLs. Accordingly, knockdown of the UGCG impacts all other glycosylated sphingolipids such as LacCer, the globo-series sphingolipid *globotriaosylceramide* (Gb3), gangliosides and complex GSLs, which are lower in its concentration in MCF-7 UGCG knockout cells (Fig. 1).

Disruption of the UGCG in mice epidermis, causes alterations in the skin lipid composition leading to the loss of the barrier function and to death due to dehydration [14]. GlcCer is needed for proper lamellar body formation and regular metabolism in the stratum corneum, which is essential for maintaining water permeability function. *Amen* et al. showed that an inducible UGCG knockout in mice keratinocytes leads to a loss of protein-bound ceramides resulting in the same fate, that occurs to the constitutive knockout mice [15].

Beside its essential functions especially in the maintenance of the epidermal water barrier function, UGCG is overexpressed in several cancer types for example in metastatic breast cancer tissue leading to poor patient prognosis [16] (Fig. 4). *Gouazé* et al. showed that this is not a unique phenomenon for breast cancer cells, because several other cancer cells e.g. human leukemia and colon cancer cells, also exhibit a high UGCG gene expression [17]. This overexpression correlates with increased *multidrug resistance protein 1* (*MDR1*) gene expression, which encodes the protein *P-glycoprotein 1* (P-gp) (*also ATP-binding cassette sub-family B member 1*, ABCB1), leading to multidrug resistance for example against vinblastin (human cervix carcinoma KB-3-1 cells; Hela derivatives), vincristine (human leukemia (HL)-60 cells) and adriamycin (human colon cancer cells SW620). UGCG blockage resensitizes multidrug resistant breast cancer cell to anticancer drugs via downregulation of *MDR1* [18].

The resistance of tumors to chemotherapeutic agents occurs via highly complex mechanisms and is a serious challenge in cancer therapy. The P-gp protein expressed at the plasma and Golgi membrane plays an important role in multidrug resistance by transporting toxic substances out of the cell (reviewed in [19]) (Fig. 4). This efflux pump, which is also expressed in normal tissue for protection from toxic substances, transports hydrophobic substances like nonylphenol ethoxylate (component of human urine) (reviewed in [20]). In addition, the P-gp protein transports hydrophobic substances like anthracyclines, taxanes and kinase inhibitors. P-gp is regulated by a variety of mechanisms on transcriptional and post-transcriptional level like *extracellular-signal regulated kinase* (ERK) signaling, *Skp1-Cullin1-FBX015* (SCF^FBX15^) *complex* and *glycogen synthase kinase 3* (GSK-3), which transcriptionally inhibits *MDR1* through *wingless* (Wnt)/β-catenin signaling (reviewed in [19]), but also via ubiquitin-proteasomal degradation by action of the *ubiquitin-activating enzyme E2 R1* (UBE2R1). For more detailed information of P-gp please see the following reviews: [21, 22].

The UGCG provides a major route for ceramide clearance in the case of an increased activity of CerS6, which generates the pro-apoptotic C_16:0_-Cer, accordingly, ceramide induced apoptosis is not induced. It is likely, that in the case of extrinsic and intrinsic apoptosis pathway the UGCG is not capable to clear the ceramide due to its localization. Through extracellular signals like oxidative stress or *Tumor necrosis factor α* (TNFα) cellular ceramide concentration increases and induces apoptosis. Ceramides can either be generated by the activation of the sphingolipid de novo synthesis, which occurs in the endoplasmic reticulum or by induction of the salvage pathway e. g. by hydrolysis of sphingomyelin. Initiation of the extrinsic apoptosis signaling pathway, occurs by ligand binding to the *Tumor necrosis factor receptor 1* (TNFR1, death receptor). Subsequently, receptor trimerization and activation of the protein *Factor associated with activation of neutral sphingomyelinase* (SMase) (FAN) that binds to the intracellular *neutral SMase domain* (NSD), thereby activating the neutral SMase, occurs. The hydrolysis of sphingomyelin in the plasma membrane leads to C_16:0_-ceramide release, which activates caspase-dependent apoptosis (reviewed in [23]). The UGCG seems not to be capable to logistically reach the ceramide for clearing it into less cytotoxic molecules. Also in the case of the intrinsic apoptotic pathway, which recently was shown to be mediated by a mitochondria-specific ceramide pool (reviewed in [24]), the residence of the UGCG in the Golgi apparatus seems to prohibit ceramide clearance.

## Materials and methods

### Cell culture

The human breast adenocarcinoma cell line MCF-7 were purchased from the Health Protection Agency (catalogue number: 86,012,803, European Collection of Cell Cultures, ECACC, Salisbury, UK) and was cultured in Dulbecco’s Modified Eagle Medium (DMEM) containing high glucose, no phenol-red and no HEPES. 1% GlutaMAX, 1% sodium pyruvat and 5% charcoaled fetal bovine serum (FBS) (Sigma-Aldrich, Deisenhofen, Germany) was added. Cells were incubated at 37 °C in an atmosphere containing 5% CO_2_. For selection of stably transfected cells, G418 (Thermo Fisher Scientific, Waltham, Massachusetts, USA) was added.

### Stable transfection of UDP-glucose ceramide glucosyltransferase (UGCG) expression plasmid in MCF-7 cells

UDP-glucose ceramide glucosyltransferase (UGCG) expression plasmid (pCMV6-ENTRY vector) was purchased from OriGene Technologies Inc. (Rockville, USA). The pTarget empty vector was purchased from Promega GmbH (Mannheim, Germany). Stable transfection was performed with Lipofectamine® 2000 Reagent (Invitrogen by Life Technologies) according to the manufacturer’s protocol. MCF-7 cells were transfected with 2 μg of the distinct plasmid and selected over 5 weeks with different G418 concentrations. Under a G418 concentration of 400 μg/ml all untransfected cells died, subsequently, this concentration was considered to be sufficient for culturing the stable transfected cells.

### Sphingolipid concentration determination by high-performance thin-layer chromatography (HPTLC)

All chemical reagents were of analytical grade or higher. Lipid standards where from Avanti Polar Lipids (Alabaster, USA) or Matreya LLC (Pleasant Gap, USA). Organic solvents were from Rathburn Chemicals Ltd. (Walkerburn Scotland). The high performance thin-layer chromatography (HPTLC) silica plates were from Whatman, UK. 9 × 10^6^ MCF-7 cells were scraped in 1 x PBS, centrifuged and frozen in liquid nitrogen. Cells were dried under the hood for three days and stored in a freezer (− 80 °C) until analyzed. Each dried sample was solubilized in ice cold MQ-water (1 ml) by bath sonication (ice-water bath) for 5 min. The solubilized samples were transferred to clean glass test tubes (with screw-caps). Total lipids were extracted by a modified Blight and Dyer protocol: 1 ml of MQ-water was added to each sample (2 ml of total water volume), 3 ml of chloroform:isopropanol (2:1, vol:vol) was added to each sample, samples were vortexed thoroughly for 10 s, and rotated end-over-end for 20 min at RT. The samples were centrifuged at 4.000 rpm for 20 min at RT to separate the phases. The organic phase (bottom) was carefully extracted with a glass Pasteur pipette and placed in a clean glass tube, without disturbing the protein precipitate at the interphase. The chloroform:isopropanol (2:1, vol:vol) extractions were repeated once, as described above, and combined with the previous extracts. 3 ml of hexane was then added to each sample, samples were vortexed, rotated and centrifuged as described above. The hexane phases (top) were extracted and combined with the previous chloroform:isopropanol extracts. The organic solvent containing the extracted lipids was dried under a stream of nitrogen. The dry samples were stored at − 20 °C. Each of the dried lipid sample was re-solubilized in an appropriate volume of chloroform:isopropanol (2:1, vol:vol). The lipid samples were normalized according to their corresponding protein concentrations and applied on an HPTLC plate using an autosampler. The HPTLC plate was developed using the solvent system chloroform:methanol:acetone:acetic acid:water (10:2:4:2:1). The sugar residues of the glycolipids were visualized by spraying the plate with an orcinol solution (0.3% orcinol in 20% sulphuric acid) and by heating (< 5 min, 120 °C). Lipids were identified with the help of commercial standards that were run in parallel with the samples on the HPTLC plate.

The precipitated protein was isolated as follows: 1 ml of chloroform and 1.5 ml of methanol was added to the remaining aqueous phases of the samples. Samples were briefly vortexed and centrifuged at 4.000 rpm for 20 min. The aqueous upper phase was carefully removed with a glass Pasteur pipette and discarded, leaving the interphase (with the precipitated proteins) untouched. 2 ml of methanol was added to each of the samples. Samples were again briefly vortexed and centrifuged at 4.000 rpm for 20 min, to pellet the precipitated proteins. The chloroform:methanol solution was carefully removed and discarded with a glass Pasteur pipette, without disturbing the pelleted proteins. The pellets were carefully dried to completion, under a stream of nitrogen. Each protein sample was re-solubilized in 500 μl of an 8 M urea solution (8 M urea in PBS, pH 6.8, 0.5% SDS) by vigorous vortexing at RT. The protein samples were stored at − 20 °C. The concentrations of the solubilized proteins were determined by the method of Lowry.

## Retrospective analysis

### UGCG and *MDR1*

#### Upstream effectors

##### UGCG promoter methylation

**Fig. 2:**
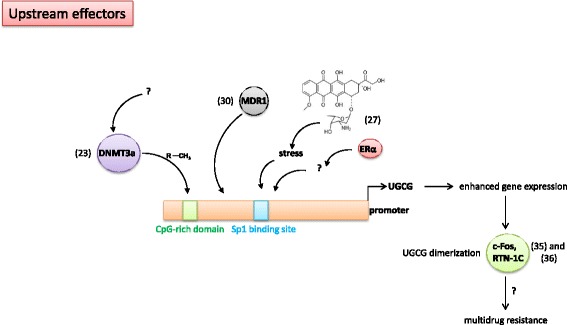
Upstream effectors – Schematic display of UGCG promoter activity regulating processes in the context of multidrug resistance. DNMT3a = *DNA (cytosine-5)-methyltransferase 3a*, CH_3_ = methyl group, MDR1 = *multidrug resistance protein 1*, ERα = *estrogen receptor α*, UGCG = *UDP-glucose ceramide glycosyltransferase*, RTN-1C = *Reticulon-1C*

Recently, Liu et al. [25] showed that the CpG island methylation at the UGCG promoter (Fig. 2) correlates inversely with its gene expression and multidrug resistance development in invasive ductal breast cancer tissue. UGCG CpG island methylation was negatively associated with estrogen receptor (ER) positive status (ERα expressing) and positively associated with HER-2 positive status [25]. In addition, in vitro experiments showed that 5-Aza-2`-deoxycytidine (decitabine; DNA methylation inhibitor) could reverse the DNA methylation status of the UGCG promoter, which restores UGCG gene expression and correlates with multidrug resistance development. The study also showed that the methylation status of the UGCG promoter is related to the expression of *DNA (cytosine-5)-methyltransferase 3a* (DNMT3a) (Fig. 2), which catalyzes the transfer of methyl groups to CpG structures in the DNA [25]. However, previous data from the same group showed a correlation of UGCG expression with ER positive and HER-2 positive status in breast cancer cells [26]. Since the DNMT3a encoding gene is frequently mutated in several cancer types [27], more studies are needed to shed light on the DNMT3a-UGCG-P-gp axes. Studies from *Raghavan* et al. show that beside UGCG, also other enzymes involved in the lipid metabolism are methylation-dependently regulated and thus leading to altered sensitivity against several drugs [28]. The cell membrane composition of doxorubicin resistant MCF-7 cells differs from membrane composition of sensitive MCF-7 cells (Fig. 4). E. g. cell membranes of resistant cells exhibit an increased sphingomyelin concentration [28]. By decitabine treatment the changes in resistant MCF-7 cell membranes could partially be reversed. The question remains whether the altered membrane composition leads to multidrug resistance induction by altering gene expression or the overexpression of the UGCG induces altered membrane composition and thus leading to multidrug resistance.

##### P-gp and others on UGCG promoter

In 2012, studies showed that doxorubicin treatment leads to an increase of UGCG mRNA and protein concentration in ER positive MCF-7 cells, but only to a slight increase in ER negative MDA-MB-231 cells [29]. This is accompanied by development of resistance to doxorubicin treatment. When the transcription factor Sp1, which has a binding site at the UGCG promoter, is inhibited by Sp1 decoy ODNs transfection, doxorubicin does not lead to an increased UGCG mRNA and protein expression in MCF-7 cells (Fig. 2). The doxorubicin resistance is reversed and apoptotic events occur. Additionally, blocking the ER subtype α (ERα) also prevents UGCG upregulation after doxorubicin treatment (Fig. 2) [29]. In ER positive MCF-7 cells blocking of ERα has a severe impact on the cell metabolism, since ERα promotes cell proliferation by direct transcriptional upregulation of several proliferation promoting genes like *Myc*, *B-cell lymphoma 2* (*BCL2*) and *E2F transcription factor 1* (*E2F1)* and indirectly like *FOS* (reviewed in [30, 31]). This led us to suggest, that the decrease of UGCG mRNA after ERα inhibition and induction of apoptosis after doxorubicin treatment may be based on an entire block of the cell metabolism. In addition, although in the study from *Zhang* et al. Sp1 decoy ODNs were used, which contain the sequence of the UGCG putative Sp1/GC-rich binding site, it cannot be excluded that another Sp1-regulated gene is blocked in its expression, which could be responsible for the UGCG promoter activity increase. If this is the case, the altered UGCG promoter activity is only a downstream effect and not directly mediated by Sp1. The same might be true for the ERα effect. It is also possible that additional transcription factors to Sp1 are involved in UGCG promoter activity regulation. Therefore, it would be helpful to perform reporter gene studies with deleted transcription factor binding sites of the UGCG promoter to verify this result. In addition, DNA-protein interactions of the UGCG promotor sequence and e.g. Sp1 should be monitored by chromatin immunoprecipitation (ChIP) assays or by *electrophoretic mobility shift assay* (EMSA) to proof the interaction. Furthermore, it has to be clarified how the doxorubicin effect on the UGCG promoter is exactly mediated since this chemotherapeutic agent leads to cell stress by induction of reactive oxygen species (ROS) or p53 accumulation and subsequently induces apoptotic signaling pathways. However, since doxorubicin is a known inducer of P-gp it has to be clearified whether or not this induction is mediated via a doxorubicin-dependent activation of UGCG gene transcription resulting in an UGCG-regulated *MDR1* gene expression (Fig. 4). Whether or not this mechanism is possible for other P-gp inducer or inhibitors such as verapamil or ciclosporin should be investigated in the future.

**Fig. 3:**
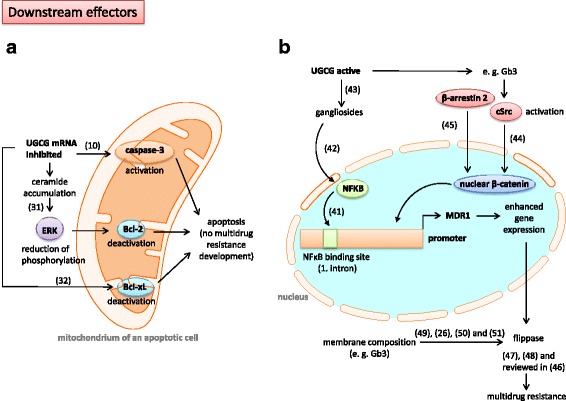
Downstream effectors – Schematic display of UGCG regulated processes in the context of multidrug resistance. **a** UGCG mRNA inhibition leads to induction of apoptotic processes and prevents multidrug resistance development. **b** UGCG activity derived GSLs such as gangliosides regulate MDR1 promoter activity. UGCG = *UDP-glucose ceramide glycosyltransferase*, ERK = *extracellular-signal regulated kinase*, Bcl-2 = *B-cell lymphoma 2*, Bcl-xL = *B-cell lymphoma-extra large*, NFκB = *nuclear factor* (*NF*) *κB*, cSrc = *tyrosine kinase*, Gb3 = *Globotriasoylceramide*, MDR1 = *multidrug resistance protein 1*

Most studies show an UGCG-dependent regulation of *MDR1* in the context of multidrug resistance induction (Fig. 3). However, studies by *Zhang* et al. revealed that this dependency occurs possibly vice versa, meaning that P-gp can directly influence UGCG expression in breast cancer cells [32] (Fig. 2). *Zhang* et al. showed that treatment of adriamycin resistant MCF-7 cells, which exhibit an increased *MDR1* and UGCG expression as compared to control cells, with siRNA against MDR1 results in decreased MDR1 and UGCG mRNA expression and protein concentration indicating that *MDR1* regulates UGCG expression. How exactly this UGCG upregulation is accomplished by *MDR1* has not been investigated.

### Downstream effectors

#### Induction of apoptosis and potential heterodimerization of UGCG

**Fig. 4:**
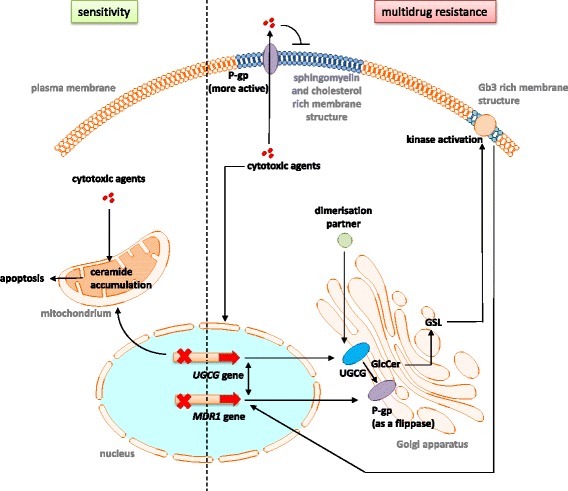
Summary of the molecular mechanisms regarding the connection between UGCG and *MDR1* in the process of multidrug resistance development. In sensitive cells (green) cytotoxic agents lead to apoptosis by, amongst other mechanisms, UGCG gene expression inhibition and following ceramide accumulation. During the process of multidrug resistance development (red) cytotoxic agents may lead to an increased gene expression of *UGCG* and *MDR1* resulting in increased UGCG protein concentration and P-gp concentration. P-gp functions as an efflux transporter for the cytotoxic agents and works also as a GlcCer flippase in the Golgi apparatus supporting ceramide and GlcCer clearance. The increased GSL production leads to alteration of raft structures in the plasma membrane resulting in increased P-gp and kinase activity. The kinases induce signaling pathways, which lead to increased *MDR1* gene transcription. Besides, the altered membrane composition blocks the diffusion of extracellular cytotoxic agents. MDR1 and UGCG are able to influence each other on transcriptional level. The influence of dimerization partners of UGCG in the context of multidrug resistance development is unknown. UGCG = *UDP-glucose ceramide glycosyltransferase*, MDR1 = *multidrug resistance protein 1*, P-gp = P-glycoprotein, GSL = Glycosphingolipid, Gb3 = Globotriasoylceramide

Most studies published in the last two decades, which investigated the involvement of the UGCG and *MDR1* in multidrug resistance, have focused on the association of UGCG and its downstream effectors. Progress was made regarding the elucidation of processes following UGCG inhibition, especially processes leading to an induction of apoptotic signaling pathways. In 2010 already, *Sun* et al. showed that inhibition of the UGCG leads to high ceramide levels in breast cancer cells and results in caspase-3 activation and subsequently cell death (Figs. 3a and 4). This study also demonstrated that in a mice xenograft model treatment with shRNA against the UGCG leads to decreased tumor growth and that beside the UGCG also the *MDR1* gene expression and subsequently P-gp protein is inhibited in vivo [12]. Years later *Wang* et al. showed that UGCG knockdown decreases the mRNA and protein levels of Bcl-2 (anti-apoptotic) via reduction of ERK phosphorylation, which was induced by ceramide accumulation in K562/A02 cells [33] (Fig. 3a). Beside Bcl-2, also *B-cell lymphoma-extra large* (Bcl-xL), which has an anti-apoptotic effect by preventing e. g. cytochrome c release from mitochondria, is decreased in its expression when the UGCG is inhibited [34] (Fig. 3a). However, *Liu* et al. published that MCF-7 cells, which overexpress the UGCG are resistant against adriamycin and ceramide toxicity, but P-gp was not detectable [35]. In addition, the phosphorylation status of the anti-apoptotic Bcl-2 protein was not altered indicating that the multidrug resistance in these cells is independent from both, *MDR1* and Bcl-2. It is possible that the sphingolipid signaling pathway is the main factor in this process, because the UGCG overexpressing cells showed only a slight increase in GlcCer amount (see also Fig. 1). This may be due to a low substrate supply or low UGCG activity, a fast processing of GlcCer to complex GSLs or degradation of GlcCer. Furthermore, the UGCG can interact with other unknown proteins and induces thereby the drug resistance. It was already shown that UGCG protein from rat is organized as a heterodimer or heterooligomer [36]. Approved binding partners for the UGCG are c-Fos [37] and the neuroendocrine-specific protein *Reticulon-1C* [38], whereby the UGCG activity does not depend on complex formation with these proteins (Figs. 2 and 4). Future studies should investigate the influence of these complexing partners in the process of multidrug resistance. The idea of UGCG protein interaction with another protein could be supported by a study showing that curcumin inhibits UGCG activity in drug-resistant cells only. This means that the multidrug resistant cells express proteins, which mediate the curcumin-induced UGCG inhibition [39]. In Hela cells only the combination of mifepristone (progesterone receptor antagonist) and mitomycin c (DNA synthesis block) inhibited UGCG [40], meaning that after mitomycin c treatment one or more specific genes are not transcribed, which are essential for induction of multidrug resistance. It is possible that also after curcumin treatment a potential dimer partner of UGCG is not expressed anymore. However, curcumin does also induce necroptosis and is thereby able to revert multidrug resistance [41]. A possible partner of UGCG protein interaction could be the cellular tumor antigen p53. Inhibition of the UGCG by *thre-1-Phenyl-2-decanoyl-amino-3-morpholino-1-propanol hydrochloride* (PDMP) or siRNA treatment in p53 deficient human osteosarcoma cells (U20S) leads to increased sensitivity to mitomycin c resulting in ceramide accumulation, decreased cell viability and apoptosis induction shown by increased caspase 3 and 7 activity [42]. p53 deficiency in U2OS cells was accomplished by stable transfection with the E6 oncoprotein of Human Papillomavirus 16 (HPV16), which mediates accelerated degradation of p53. The data of Haynes et al. may indicate a p53-dependent UGCG regulation (Fig. 2), but studies with p53 proficient cells should be performed in the future. Another interesting target protein for UGCG interaction would be the P-gp protein itself. Furthermore, it has to be clarified in what kind of way the UGCG-derived GSL are involved in apoptotic processes since ceramides induce apoptosis via membrane bound receptors.

#### Glycosphingolipids on *MDR1*

A few studies describe the direct influence of UGCG activity on *MDR1* expression, indicating that also gangliosides resulting from UGCG activity may have an effect on *MDR1*. These studies are described in this chapter. It could be shown that inhibition of the transcription factor *nuclear factor* (NF) ΚB results in decreased *MDR1* gene and P-gp protein expression [43]. Thereby, NFΚB seems to bind directly to the *MDR1* promoter through an intronic site (Fig. 3b). In this respect, it is interesting that a ganglioside-dependent activation of NFΚB via PKC and NDAPH oxidase could be shown in microglia [44] (Fig. 3b). The precursor of all gangliosides is GlcCer generated by the UGCG and accordingly an increased UGCG activity leads to increased gangliosides and/or cerebrosides levels [45] (Figs. 1 and 4). The influence of NFΚB on multidrug resistance should be also investigated in other cell types to determine whether or not this is a unique mechanism. *Liu* et al. showed for the first time that globo series GSL produced by UGCG activity mediate *MDR1* expression through cSrc and β-catenin signaling [46]. Figure 3b shows that UGCG overexpression combined with chemotherapeutic agent treatment leads to increased concentrations of e. g. Gb3 and Gb5 in GSL-enriched microdomains (GEM) resulting in tyrosine kinase cSrc activation and decreased β-catenin phosphorylation and increased nuclear β-catenin (Fig. 4). cSrc-induced phosphorylation of GSK-3β, which is inactive when phosphorylated and therefore leads no more to phosphorylation of β-catenin, results in nuclear β-catenin. The nuclear β-catenin may bind in a complex with the *T-cell factor 4* (Tcf4) to the Tcf4/*lymphoid enhancer factor* (LEF) binding motif at the *MDR1* promoter and thus enhancing promoter activity leading to increased efflux of anticancer drugs of the cell [46]. Another possibility to increase GSK-3β phosphorylation and subsequently reduce GSK-3β activity is to inhibit *protein phosphatase 2* (PP2A) activity, which dephosphorylates the GSK-3β protein leading to activation of the protein and degradation of β-catenin. However, currently, a correlation of β-arrestin 2 and *MDR1* expression could be shown in breast cancer [47]. The protein β-arrestin 2 is highly expressed in multidrug resistant cells and a knockout of β-arrestin 2 decreased *MDR1* gene expression and P-gp protein expression dramatically leading to increased doxorubicin sensitvity. Since β-arrestin 2 is able to stabilize β-catenin by interacting with the intracellular proteins Dishevelled and Axin, an increased expression leads to translocation of β-catenin to the nucleus, where the previous described mechanisms occur (Fig. 3b). In the future it has also to be investigated whether or not β-arrestin 2 is able to bind directly to β-catenin and thereby execute the multidrug resistance inducing effect.

#### P-gp as a flippase

The UGCG protein modifies ceramide on the cytosolic surface of the Golgi apparatus (Fig. 4). The conversion of GlcCer to LacCer by adding a galactose molecule is accomplished in the luminal leaflet of the Golgi apparatus. For this reaction GlcCer has to be flipped from the cytosol to the Golgi apparatus lumen. *Morad and Cabot* postulate that P-gp contributes to multidrug resistance by adopting GlcCer transfer from the Golgi apparatus cytosol to the Golgi lumen in form of a GlcCer flippase and thereby promoting ceramide clearance (reviewed in [48]. Subsequently, ceramides are not able to induce apoptotic signaling pathways anymore. However, *De Rosa* et al. showed that P-gp activity as a flippase is required for neutral GSL production, but not for acidic GSL (e. g. gangliosides) generation [49]. The hypothesis of the flippase task of P-gp is also supported by the fact that beside ceramides, GlcCer seem to induce toxic effects in the cell [50]. Cer are anabolized to GlcCer, which undergo clearance by flipping and usage for non-toxic complex GSL synthesis (Fig. 4). It is an ongoing discussion whether or not the Cer and GlcCer clearance or the production of complex GSL are the mechanisms to overcome drug sensitivity. The increased GSL production and subsequent transport to cell membranes alters P-gp activity. There are a variety of parameters, which influence the P-gp activity (reviewed in [20]). E. g. the presence of cholesterol, which alters membrane packing and fluidity affects catalytic activity of P-gp and its stimulation by substrates and modulators (Fig. 4). It is to add, that possibly the UGCG structure may allow the GlcCer to be released to the inner as well as to the outer leaflet of the Golgi apparatus. The structure of the ceramide determined by its chain length could determine a preference for the inner or outer leaflet of the cell organell. In this case the cell could discriminate between GlcCer intended for complex GSL synthesis and GlcCer taking the sphingolipid transport protein FAPP2 route to the trans-Golgi apparatus and a flippase would be unnecessary.

### Impact of UGCG activity induced alterations of membrane composition by GSL

Beside the UGCG-P-gp connection, it could be shown that the composition of the cell membrane plays an important role in the induction of multidrug resistance. The question is how UGCG activity can influence P-gp activity not directly, but via GSL synthesis, which lead to alteration of the membrane composition. *Tyler* et al. showed that the globo-series GSL Gb3 concentration is increased in non-small cell lung cancer (NSCLC) and malignant pleural mesothelioma (MPM) cells, which are cisplatin resistant [51] (Fig. 4). Treatment with the UGCG inhibitor *1-phenyl-2-palmitoylamino-3-morpholino-1-propanol* (PPMP) decreased the induced cell surface Gb3, but not P-gp protein expression (*MDR1* gene expression pattern similar). Nevertheless, Gb3 silencing restores cisplatin sensitivity, indicating that the UGCG protein does not have a direct effect on P-gp. This leads to the assumption that Gb3 alters membrane composition leading to a changed P-gp activity status. Another study already showed that membrane lipids of drug resistant MCF-7 cells exhibit an increased lipid packing and membrane fluidity as compared to membrane lipids of sensitive cells. Following decitabine treatment these biophysical changes in membrane lipids are altered meaning that the packing density is decreased and the membrane fluidity is increased even more [28]. The authors hypothesized that the resistant cells exhibit increased cholesterol-sphingomyelin raft structures, which may influence drug transport and therefore induce multidrug resistance. *Ghetie* et al. could already show that P-gp is more active in cholesterol and sphingomyelin rich raft structures as compared to a localization outside these membrane structures [52] (Fig. 4). The changes regarding the biophysical properties in the study of *Raghavan* et al. are blocked by decitabine treatment [28] indicating an epigenetic mechanism meaning methylation-dependent regulation of enzymes of the lipid metabolism in the cell as it has already been shown for the UGCG. Since decitabine inhibits the methylation of all genes in the cell, no detailed information about the molecular mechanisms are available. *Peetla* et al. reviewed in 2013 that in drug resistant cell membranes the sphingolipid and/or cholesterol content is increased leading to a higher membrane order and less permeability resulting in reduced lipophilic drug diffusion and less effective cytotoxic substances [53] independent from P-gp (Fig. 4). This shows, not only the impact on membrane proteins is altered by changed lipid composition, but also the behavior of the toxic substance in the lipid bilayer.

## Conclusions

The influence of the UGCG on multidrug resistance was investigated in depth for the last two decades. But still many pieces of the puzzle need to be analyzed. The link between UGCG overexpression and multidrug resistance induction is more complex than it was anticipated. Particularly, the influence of membrane properties should be investigated in future studies since during malignant transformation of cells the biophysical parameters of the membranes changes.

## Abbreviations

ABCB1: ATP-binding cassette sub-family B member 1
Bcl-2: B-cell lymphoma 2
Bcl-xL: B-cell lymphoma-extra large
Cer: Ceramide
CerS6: Ceramide synthase 6
ChIP: Chromatin immunoprecipitation
CRISPR: Clustered Regularly Interspaced Short Palindromic Repeats
CS: CRISPR score
cSrc: Tyrosine kinase
DNMT3a: DNA (cytosine-5)-methyltransferase 3a
E2F1: E2F transcription factor 1
EMSA: Electrophoretic mobility shift assay
ER: Estrogen receptor
ERK: Extracellular-signal regulated kinase
FAN: Factor associated with activation of neutral sphingomyelinase
FBS: Fetal bovine serum
Gb3: Globotriaosylceramide
GEM: Glycosphingolipid-enriched microdomain
*GLA*: α-galactosidase A gene
GlcCer: Glycosylceramide
GSK-3: Glycogen synthase kinase 3
GSL: Glycosphingolipid
LacCer: Lactosylceramide
LEF: Tcf4/lymphoid enhancer factor
*MDR1*: Multidrug resistance protein 1 gene
NFκB: Nuclear factor (NF) κB
NSD: Neutral SMase domain
PDMP: Thre-1-Phenyl-2-decanoyl-amino-3-morpholino-1-propanol hydrochloride
P-gp: P-glycoprotein
PPMP: 1-phenyl-2-palmitoylamino-3-morpholino-1-propanol
ROS: Reactive oxygen specie
SMase: Sphingomyelinase
Tcf4: T-cell factor 4
TNFR1: Tumor necrosis factor receptor 1
TNFα: Tumor necrosis factor α
UBE2R1: Ubiquitin-activating enzyme E2 R1
UGCG: UDP-Glucose ceramide glycosyltransferase
Wnt: Wingless
